# Effects of Sleep Quality on Melatonin Levels and Inflammatory Response after Major Abdominal Surgery in an Intensive Care Unit

**DOI:** 10.3390/molecules22091537

**Published:** 2017-09-12

**Authors:** Necdet Fatih Yaşar, Bartu Badak, Ağgül Canik, Sema Şanal Baş, Sema Uslu, Setenay Öner, Ersin Ateş

**Affiliations:** 1 Department of General Surgery, Medical School, Eskisehir Osmangazi University, Eskisehir 26480, Turkey; drbartu@gmail.com (B.B.); eates@ogu.edu.tr (E.A.); 2 Department of Biochemistry, Medical School, Eskisehir Osmangazi University, Eskisehir 26480, Turkey; aggulcanik@hotmail.com (A.C.); suslu@ogu.edu.tr (S.U.); 3 Department of Anesthesiology and Reanimation, Medical School, Eskisehir Osmangazi University, Eskisehir 26480, Turkey; drsemasa@gmail.com; 4 Department of Biostatistics, Medical School, Eskisehir Osmangazi University, Eskisehir 26480, Turkey; oners@ogu.edu.tr

**Keywords:** melatonin, intensive care unit, sleep quality, surgery, inflammatory response

## Abstract

Disruption of nocturnal sleep in an intensive care unit may remarkably affect production of melatonin, which is also known to have anti-inflammatory properties. In the present study, we aimed to investigate the effect of sleep quality on melatonin levels and inflammation after surgery. Thus, we compared the patients, who were screened in the side-rooms where the lights were dimmed and noise levels were reduced, with the patients who received usual care. Preoperative and postoperative urine 6-sulphatoxymelatonin, serum interleukin-1 (IL-1), interleukin-6 (IL-6), and c-reactive protein (CRP) levels were measured and data on sleep quality was collected using the Richards–Campbell Sleep Questionnaire. Postoperative CRP and IL-6 levels were greater in the control group than in the experimental group, whereas postoperative 24 h melatonin levels were greater than preoperative levels and the difference was steeper in the experimental group in concordance with sleep quality scores. Thus, the regulation of light and noise in ICUs may help the recovery after major surgeries in patients, potentially by increasing melatonin production, which has anti-inflammatory properties.

## 1. Introduction

Endogenous melatonin is produced by the pineal gland at night under normal conditions and regulates the sleep–wake cycle [1,2,3]. Sleep disturbances are accompanied by abnormal melatonin secretion, such as phase delay [3]. It is well known that artificial light administered at night suppresses melatonin production [3]. Medications, automatic blood pressure cuff inflation, patient care interventions, and other environmental factors such as noise may also disrupt nocturnal sleep which may disturb the endogenous rhythms in intensive care unit patients and may affect melatonin production remarkably [3,4,5].

In addition to its physiological roles in regulating sleep patterns, melatonin has been demonstrated to provide anti-inflammatory effects [3]. In experimental models, melatonin has been shown to counteract the rise in pro-inflammatory cytokine levels [6,7,8,9,10] and reduce elevated plasma CRP levels [11].

Although some previous studies have investigated the circadian pattern of melatonin in ICU patients [12] and suggested that use of earplugs and eye masks improved sleep quality and nocturnal melatonin levels in a simulated ICU environment [13]. However, to our knowledge, the effects of controlling the environmental conditions on endogenous melatonin levels in ICU has not been studied yet. In this prospective randomized study, we aimed to assess the effects of controlling noise and light in the ICU on melatonin and inflammatory response after major abdominal surgery.

## 2. Results

**Figure 1:**
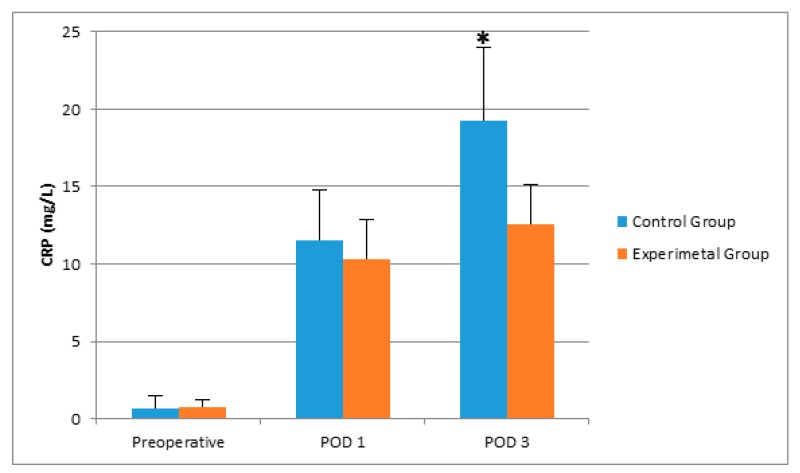
The effects of controlling noise and light in ICU on plasma CRP levels after major abdominal surgery. * *p* < 0.001 versus experimental group.

**Figure 2:**
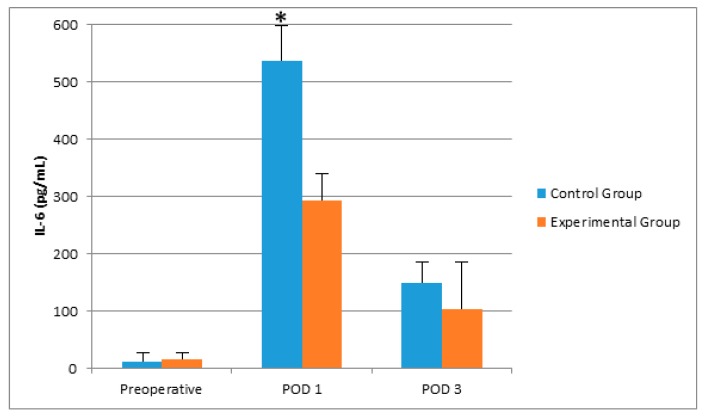
The effects of controlling noise and light in ICU on plasma IL-6 levels after major abdominal surgery. * *p* = 0.001 versus the experimental group.

**Figure 3:**
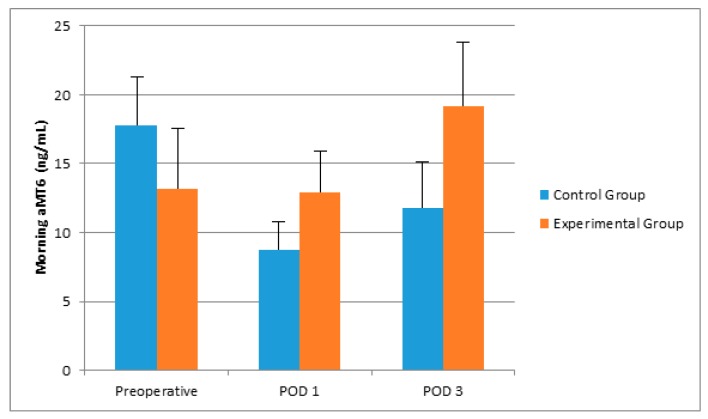
The effects of controlling noise and light in ICU on morning aMT6 levels after major abdominal surgery.

**Figure 4:**
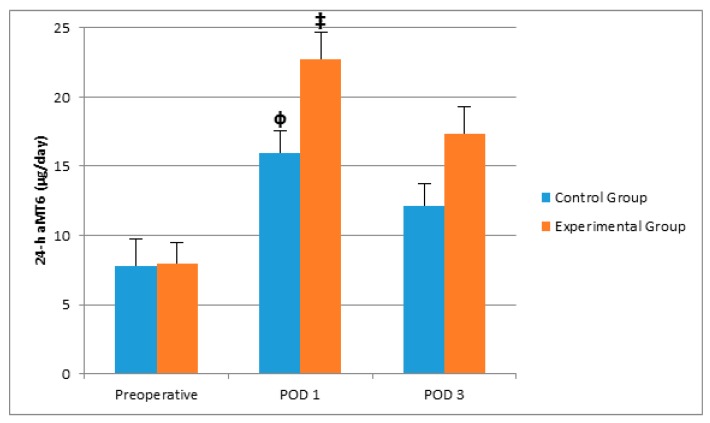
The effects of controlling noise and light in ICU on 24-h aMT6 levels after major abdominal surgery. ^Φ^ *p* < 0.05 versus preoperative levels in the control group, ^‡^ *p* < 0.05 versus preoperative levels in the experimental group.

There was no significant difference between groups with respect to age, gender, and operation durations (Table 1). Postoperative sleep quality scores were greater in the experimental group, significantly (Table 1). POD 3 CRP and POD 1 IL-6 levels were greater in the control group than in the experimental group (Table 2, Figure 1 and Figure 2). Even if the mean melatonin levels were greater in the experimental group, the difference did not reach statistical significance (Table 2, Figure 3 and Figure 4).

When we compared the preoperative, POD1, and POD3 results within the same groups, we observed that postoperative CRP and IL-6 levels were greater than the preoperative levels in the control group (*p* < 0.05). In both control and experimental groups, POD1 24 h melatonin levels were greater than preoperative levels significantly (*p* < 0.05). However, the increase in the experimental group was steeper (Figure 4).

## 3. Discussion

In the present study, we have observed that the postoperative inflammatory response was reduced in the experimental group compared to the control group, in concordance with the changes in melatonin levels and sleep quality.

As Hu et al. have suggested, we observed that controlling the environmental conditions in the intensive care unit helped for better sleep quality [13]. On the first postoperative night, the scores were higher in the experimental group, and on the third postoperative night the difference was even greater.

Major abdominal surgery leads to release of both pro-inflammatory and anti-inflammatory cytokines and the inflammation should be balanced by the anti-inflammatory process for the recovery. As anticipated, the postoperative levels of serum IL-6 and CRP were elevated, however, the elevations in IL-1 levels were not statistically different, similarly to the study of Kvarnström et al. on complement activation and inflammatory response after major abdominal surgery [14].

Shilo et al. suggested that the ICU patients lacked normal sleep behavior, which was accompanied by impairment of normal melatonin secretion [5]. In the present study, we have observed that morning melatonin secretion was impaired which was evident in the control group. Hu et al. showed that use of earplugs and eye masks improved sleep quality and nocturnal melatonin levels in a simulated ICU environment [13]. Our results have showed less impairment in the levels of POD 1 morning aMT6 levels in the experimental group. Moreover, even if the results did not show any statistical significance, it was conspicuous that POD 3 morning aMT6 levels were even higher than preoperative levels in the experimental group.

In addition to its physiological roles in regulating sleep patterns, melatonin has been demonstrated to provide anti-inflammatory effects [3]. In an experimental septic shock model, melatonin has been shown to counteract the rise in pro-inflammatory cytokine levels including IL-12, TNF-α, and interferon-γ, and to induce the production of the anti-inflammatory IL-10 [6]. Moreover, Veneroso et al. reported that melatonin reduced cardiac inflammatory injury induced by acute exercise by decreasing the mRNA levels of IL-1, IL-6 and TNF-α [7]. Similar effects of melatonin on interleukin levels in models of heat stroke, acute liver injuries, and neuroinflammation induced by methamphetamine- and oxaliplatin-induced pain, have been observed [8,9,10,15,16]. The other sites in which anti-inflammatory properties of melatonin were observed include renal, pancreatic, and even retinal tissue [17,18,19]. Furthermore, anti-inflammatory effects of melatonin in a thermal trauma model was revealed by the showing the reduction in the elevated plasma CRP levels [11]. However, in all these studies, melatonin has been applied as a treatment. However, Hu et al. showed that use of earplugs and eye masks improves sleep quality and nocturnal melatonin levels in a simulated ICU environment [20]. To our knowledge, the only study on the relation between melatonin secretion and surgical immune response has been conducted on patients who underwent coronary bypass surgery [21]. In this prospective study, it has been shown that melatonin levels in blood samples taken one hour before and four hours after the surgery, were higher in the group of patients who underwent surgery in the morning compared with the patients who were operated in the afternoon. This finding was actually consistent with the normal circadian rhythm of melatonin. Furthermore, authors have shown that, in the group with higher levels of melatonin, plasma ICAM-1 levels, an inflammatory marker, were reduced [21]. In the present study, preoperative samples were obtained two days before the surgery, which was independent from the timing of the procedure, thus, preoperative melatonin levels were similar in both groups. Postoperative 24 h melatonin levels in both groups were surprisingly higher than the preoperative 24 h melatonin levels and actually even higher in the experimental group. This may be explained by the activation of hypothalamo–hyposphyseal axis while circadian rhythm was disrupted. Nevertheless, the postoperative increase in IL-6 and CRP levels in the control group was significant while it did not reach statistical significance in the experimental group. On the other hand, even though the difference in aMT6 levels was not significant, it was consistently greater in the experimental group in both morning and 24 h urine samples on POD 1 and POD 3. This was in concordance with the anti-inflammatory response and consistent with the findings of the previously-mentioned study.

The study has a number of limitations which should be reviewed. The melatonin secretion profile displays a great inter-subject heterogeneity [3] and our sample sizes were small, which limited the power of the statistical analysis. Actually, this might be the reason why the difference in melatonin levels did not reach significance. Therefore, we also compared the preoperative and postoperative levels of the parameters in both groups separately. Another limitation was that the environmental stimuli in the ICU may be varied depending on the number of the patients and the workload.

## 4. Materials and Methods

This study has been conducted in accordance with the Helsinki Declaration and has been approved by Eskisehir Osmangazi University Ethics Committee (No. 2016-1309, NCT02824770). Forty consecutive patients, aged 18–65, who underwent elective major abdominal operations were enrolled in the study after obtaining informed consent. Patients with any evidence of inflammatory diseases during postoperative care, as well as patients who were on any hormone replacement therapy before surgery and who underwent surgery due to any endocrine neoplasms, were excluded. All patients received the usual postoperative pain management including continuous infusion of Bupivacaine (Bustesin^®^, Istanbul, Turkey) with the Pain Buster^®^ system (Lake Forest, CA, USA) into the wound at a rate of 5 cc/h and infusion of Tramadol HCl (Tramosel^®^, Istanbul, Turkey) via patient-controlled intravenous analgesia (PCA) in the surgical intensive care unit. The patients were randomly assigned to either the experimental group or the control group. The control group received the usual care. The patients in the experimental group were screened in the side-rooms where normally the patients who either have infections or are at risk of infection, are nursed. The study intervention included dimming the lights to 40 lux and closing the doors of the side-room to decrease the noise level below 40 dB between 11 p.m. and 5 a.m.

Preoperative blood and urine samples were collected two days before the operation. Duration of the surgery, gender, and age of the patients were recorded. After the operation, blood and urine samples were collected on postoperative day (POD) 1 and day 3. Melatonin production was monitored by determination of urine 6-sulphatoxymelatonin (aMT6s) which is a reliable indicator of circulating melatonin [12]. Due to the diurnal secretion, both 24 h and spot morning urine at 7:00 a.m. were collected. Along with urine aMT6, serum interleukin-1 (IL-1), interleukin-6 (IL-6), and c-reactive protein (CRP) levels were measured before surgery and on PODs 1 and 3. Data on sleep quality was collected using the Richards–Campbell Sleep Questionnaire [20].

Urine levels of aMT6 were determined by Enzyme-Linked Immunosorbent Assay (ELISA) using IBL International GmbH ELISA kit (Hamburg, Germany). Serum levels of IL-1 and IL-6 were analyzed by ELISA and Enzyme Amplified Sensitivity Immunassay (EAISA), respectively, using a DIAsource kit (Louvain-la-Neuve, Belgium) and CRP levels were determined by automated analytical system (Siemens Dimension Vista^®^ 1500, Siemens Healthcare Diagnostics, Tarrytown, NY, USA).

### Statistics

In the present study, normality of continuous variables was evaluated with a Shapiro–Wilk test. Comparisons between the experimental and control groups were conducted with independent samples *t*-tests for normally-distributed variables, which were age, gender, postoperative CRP levels, POD 1 IL-1 and 24-h urine aMT6 levels, and Richards–Campbell sleep quality scores. Mann–Whitney U test was used for the comparison of non-normally distributed variables, which were operation durations, preoperative CRP levels, IL-1, IL-6, morning, and 24-h urine aMT6 levels, POD 1 IL-6, morning urine aMT6, and POD 3 IL-1, IL-6, morning, and 24-h urine aMT6 levels, and Richards–Campbell sleep quality scores. Descriptive statistics of normally-distributed variables were given as mean ± standard error, while median and 25–75% percentile values were given for non-normally-distributed variables. Since the variables were not normally distributed, the difference between preoperative, POD 1, and POD 3 in both control and experimental groups were evaluated with Friedman’s two-way analysis of variance.

## 5. Conclusions

As a result, regulation of light and noise in ICUs may increase the sleep quality in ICU and may help with recovery after major surgeries in the patient by reducing the inflammatory response and, possibly, may help the increase in the melatonin levels. Future studies should be conducted to show the relevance between the surgical stress and melatonin secretion.

## Figures and Tables

**Table 1:** Basic patient demographics, duration of surgery, and postoperative Richards–Campbell sleep quality scores.

	**Control Group Mean ± S.Error**	**Experimental Group Mean ± S.Error**	***p***
Age	55.50 (±1.74)	53.35 (±1.91)	0.410
Gender (number of males)	16	15	
Richards–Campbell sleep quality scores POD1	41.65 (±1.60)	49.95 (±1.56)	0.001
	**Median (25–75%)**	**Median (25–75%)**	
Operation duration	3.50 (2.50–4.75)	3.00 (2.50–3.88)	0.602
Richards–Campbell sleep quality scores POD3	46.50 (43.00–52.50)	60.50 (52.75–62.75)	<0.001

**Table 2:** Preoperative and postoperative plasma CRP, IL-1, IL-6, and urine aMT6 levels.

	Control Group Mean ± S.Error/ Median (25–75%)	Experimental Group Mean ± S.Error/ Median (25–75%)	*p*
Preoperative			
CRP (mg/L)	0.66 (0.34–1.84)	0.78 (0.34–1.35)	0.820
IL-1 (pg/mL)	8.54 (2.79–33.75)	14.79 (6.23–30.56)	0.355
IL-6 (pg/mL)	12.47 (8.33–26.95)	16.29 (8.86–31.03)	0.529
Morning aMT6 (ng/mL)	17.76 (4.40–25.18)	13.17 (7.60–30.56)	0.989
24-h aMT6 (µg/day)	7.82 (3.97–16.56)	7.99 (4.60–11.76)	0.947
POD 1			
CRP (mg/L)	11.51 (±0.99)	10.28 (±0.74)	0.330
IL-1 (pg/mL)	25.70 (±3.53)	19.10 (±2.62)	0.141
IL-6 (pg/mL)	537.31 (282.26–678.15)	293.24 (195.28–454.44)	0.013
Morning aMT6 (ng/mL)	8.70 (4.76–16.64)	12.91 (7.81–26.68)	0.211
24-h aMT6 (µg/day)	15.92 (±2.22)	22.73 (±3.52)	0.112
POD 3			
CRP (mg/L)	19.28 (±1.35)	12.56 (±1.02)	<0.001
IL-1 (pg/mL)	23.55 (4.68–39.77)	17.15 (5.40–37.26)	0.883
IL-6 (pg/mL)	148.93 (87.35–175.10)	103.97 (83.70–143.66)	0.183
Morning aMT6 (ng/mL)	11.78 (5.91–34.78)	19.14 (10.13–29.38)	0.383
24-h aMT6 (µg/day)	12.10 (7.89–21.38)	17.34 (7.58–34.70)	0.327
